# Successful use of next generation genomic sequencing (NGS)-directed therapy of clear cell carcinoma of the ovary (CCCO) with trametinib and metformin in a patient with chemotherapy-refractory disease

**DOI:** 10.1186/s40661-015-0013-2

**Published:** 2015-08-28

**Authors:** Michael P. Castro, Bradford P. Whitcomb, Deborah A. Zajchowski, Robert L. Coleman

**Affiliations:** Personalized Cancer Medicine PLLC, 1329 Lusitana St. Suite 609, Honolulu, HI 96813 USA; Division of Gynecologic Oncology, Tripler Army Medical Center, TAMC, Honolulu, HI USA; The Clearity Foundation, 4365 Executive Drive Suite 1500, San Diego, CA 92121 USA; Department of Gynecologic Oncology & Reproductive Medicine, Clinical Research Programs, University of Texas MD Anderson Cancer Center, 1155 Herman Pressler Dr., CPB 6.3590, Houston, TX 77030 USA

**Keywords:** Ovarian cancer, Clear cell carcinoma, Molecular profiling, Targeted therapy, PIK3CA mutation, KRAS mutation, Trametinib, Metformin, Precision medicine, Personalized medicine, Genomic, Next generation sequencing

## Abstract

**Purpose:**

Compared to other subtypes of epithelial ovarian cancer, clear cell carcinoma of the ovary bears an ominous reputation for chemotherapy resistance, increased relapse rate, and diminished survival. Among patients with distinct histopathologic subtypes, molecular analyses have identified a variety of known drivers of the malignant behavior, and depict a striking heterogeneity.

**Methods:**

A patient with rapidly metastatic CCCO that was refractory to taxane, platinum, pemetrexed, and bevacizumab-based strategies underwent molecular profiling which disclosed dual MAPK and PI3K/AKT/mTOR pathway mutations.

**Results:**

Combined targeted therapy with trametinib and metformin resulted in a dramatic disease regression without toxicity.

**Conclusion:**

The case highlights the utility of precision medicine combining individual molecular diagnosis with rational therapeutic intervention with targeted agents.

## Background

Clear cell carcinoma of the ovary (CCCO) represents a distinct histopathologic subtype [1] comprising 3.7–12.1 % of epithelial ovarian carcinoma (EOC). In general, CCCO has earned notoriety for being a particular challenge for management characterized by higher recurrence rates among patients with early stage disease, poor responsiveness to chemotherapy, especially platinum [2–7], *de novo* drug resistance, and inferior survival compared to other subtypes of EOC [8]. In light of these characteristics, many investigators have opined that CCCO deserves a unique treatment strategy as a distinct disease entity. Nevertheless, current guidelines recommend similar adjuvant treatment regimens as used for other EOC histologies.

Molecular studies have demonstrated differences in the genomic characteristics of CCCO compared to other histological subtypes of EOC and suggest it may be amenable to different therapeutic approaches. In addition, individual differences have been observed between patients despite the common clear cell histology. In a recent study of 69 CCCO’s patients analyzed by next generation sequencing (NGS), PIK3CA was the most common mutation (52 vs. 8 % in all EOCs and 14 % in mixed CCCO’s), followed by TP53 (16 %) and KRAS (11 %). Mutations in FBXW7 (10 %), APC (7 %), and ATM (6 %) were observed at a higher rate than in other EOCs. Among 33 with PIK3CA mutations, 4 (12 %) had co-existing mutations in KRAS and 2 (6 %) had TP53 mutations while 70 % (23/33) over-expressed cMET and 12 % had a loss of PTEN [9]. Relative cyclin E mRNA expression is often significantly higher in the CCCO’s [10], Hepatocyte nuclear factor-1 beta has also been identified as a molecular marker and touted as a possible molecular target for intervention [11].

We describe the application of DNA sequencing to identify known molecular drivers of malignancy in a patient with chemotherapy-refractory metastatic CCCO. The use of off-label, NGS-directed therapy led to meaningful disease regression and control.

## Case presentation

The patient is a 40-year old woman with a history of endometriosis and infertility. In the spring of 2014 she developed pelvic pain. Trans-vaginal ultrasound revealed endometriosis and a complex cystic mass of the right ovary consistent with possible malignancy. The CA-125 was 32. She was taken to the operating room for a laparoscopic right ovarian cystectomy. Based on visible tumor within the ovary at surgery, the operation was converted to a staging laparotomy with traditional cytoreductive surgery with total abdominal hysterectomy, bilateral salpingo-oophorectomy, bilateral pelvic and para-aortic lymph node dissection, peritoneal biopsies, and peritoneal washings. No residual disease remained after surgery. Pathology disclosed clear cell carcinoma of the right ovary. Examination of the uterus revealed adenomyosis and endometriosis with focal clear cell carcinoma near the parametrial margin; 13 pelvic and para-aortic lymph nodes from the right and 15 from the left side showed no evidence of metastatic disease. The omentum contained a small focus of malignant cells, however the remainder of samples from the pelvis, paracolic gutters, and the undersides of the diaphragms revealed no cancer. She was staged as having FIGO stage IIIA2 clear cell carcinoma of the ovary.

Beginning in June 2014 she was treated with post-operative, adjuvant, dose dense paclitaxel and carboplatin x 6 cycles, which were completed in October 2014. A post-treatment PET scan in October 2014 revealed metastatic progression with hypermetabolic metastatic disease in the liver and multiple sites of hypermetabolic lymphadenopathy throughout the pelvis including the vaginal cuff. Chemotherapy utilizing pemetrexed and bevacizumab was administered the following week for three treatment cycles until December 2014. In January 2015, another PET scan disclosed new hypermetabolic disease in the left lung measuring 8 mm, additional new hypermetabolic lymph nodes in the left iliac chain, and progression in the liver with multiple metastases.

Germline BRCA1 and two testing at Myriad Genetics was negative. Comprehensive genomic analysis of the primary ovarian tumor tissue was arranged by the Clearity Foundation (www.clearityfoundation.org) and included NGS analysis at Foundation Medicine, Inc. (Cambridge, MA) to identify actionable genomic alterations in key oncogenes and tumor suppressor genes (exonic regions of 315 genes). This analysis revealed KRAS, PIK3CA, and TERT mutations (Table 1).

**Table 1:** FoundationOne™ genomic testing identified three driver mutations, of which two were actionable with available drugs

Therapeutic Implications		
Genomic Alteration	FDA-approved therapies for ovarian cancer	FDA-approved therapies for other tumor types
KRAS	None	Trametinib
G12v		
PIK3CA	None	Everolimus, temsirolimus
H1047R		
TERT	None	None
Promoter 124 C > T		

The MEK inhibitor, trametinib (2 mg daily) and metformin (850 mg q12hr) which activates AMPK and also inhibits the AKT-mTOR pathway, were instituted 3 weeks later. The patient was not a participant on any active research protocol. Trametinib was obtained through Tripler Medical Center which does not restrict the use of off-label medicine or require insurance pre-authorization. The selection of metformin in lieu of a standard mTOR inhibitor, such as everolimus, was made because of the failure of the phase I effort to define a safe combination of trametinib and everolimus for phase II testing [12].

**Fig. 1:**
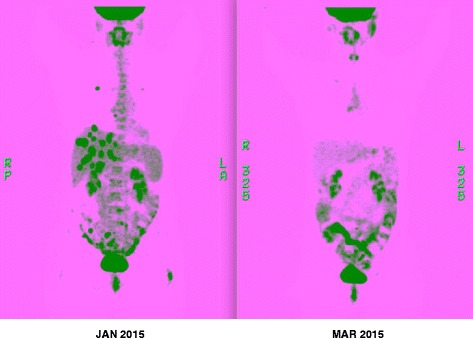
Comparison of baseline (*left*) and post-treatment (*right*) PET scans (3D MIP images)

At the start of treatment, the CA125 was 936. After 2 months of treatment, the CA125 fell to 69. A follow-up PET scan at that time revealed complete resolution of the metastatic disease in the liver and lung, fading and nearly complete disappearance of the vaginal cuff lesion, disappearance of pelvic adenopathy, and a new hypermetabolic focus at the aortic bifurcation (Fig. 1). The patient experienced no side effects or clinical toxicities from the treatment. Hypoglycemia did not occur. The response lasted 5 months before progressive disease ensued. Repeat tissue sampling to determine the mechanism of resistance is under consideration.

## Conclusions

This case of clear cell carcinoma of the ovary depicts the notorious problems of chemotherapy resistance and early relapse. In just 6 months, her outlook evolved from a hope for cure to imminent death with tumor growth on both adjuvant taxane-platinum and pemetrexed-bevacizumab combinations. Her cancer possesses a PIK3CA mutation, a finding in 34 % of CCCO (http://cancer.sanger.ac.uk/cosmic/search?q=PIK3CA+ovarian), causing upregulation of the AKT pathway, which is frequently associated with *de novo* resistance to platinum combinations [13]. In the sister disease, clear cell carcinoma of the kidney, molecular aberrations in the AKT pathway have also been reported in 20 % of patients [14], and have been successfully treated with MTOR inhibitors. A KRAS mutation was also identified in this patient’s cancer, a finding present in 14 % of CCCO patients [15]. These mutations suggested a dual strategy of MEK and AKT pathway inhibition.

Derangement in the MAP kinase pathway plays a central role in the proliferation and progression of many cancers. Trametinib is an FDA-approved allosteric inhibitor of MEK1 and MEK2 capable of inhibiting cancers with KRAS mutations by producing downstream blockade. Metformin has been shown to alter AMPK, a protein kinase that directly and indirectly regulates mTOR signaling via phosphorylation of Raptor and TSC2, respectively. Proof of concept studies of metformin have been reported, including regression of breast [16] and pancreatic cancers [17]. This approach, employing simultaneous blockade of AKT and MAPK pathways, has produced durable responses [18, 19]. In patients with KRAS-mutant ovarian cancer, MEK and PI3K inhibitors have provided response rates of 50–75 % [20–22]. Additionally, KRAS G12V mutation, which was present in this case, is a marker for responsiveness to MEK inhibition in patients with low grade serous ovarian cancer [23]. Even though efforts to combine MEK inhibitors with PI3K/AKT/m-TOR inhibitors have thus far been plagued by the inability to identify a tolerable, safe dose for phase II testing [12], remarkably this patient experienced no toxic effects from the combination of trametinib and metformin.

This patient’s success validates the strategy of dual pathway inhibition as an approach to cancer with mutations in both the MAPK and AKT pathways. It highlights the potential safety of combining metformin and trametinib. This case also demonstrates the utility of linking molecular diagnosis with therapeutic decision-making for patients with advanced, chemotherapy-refractory disease. As such, the use of precision medicine to provide driver-drug strategies offers hope for patients with CCCO and other malignancies with this molecular etiology. This process is a promising, rational method for replacing the genomically-naïve approach to treatment that still dominates the standard of care for the entire spectrum of epithelial ovarian cancer.

This patient was fortunate to gain access to a drug that is usually unavailable to ovarian cancer patients due to strict payor policies that follow the drug license to the letter. However, over-zealous regulatory behavior and wholesale restriction of physician prescribing can deprive patients of life-saving medicines. In an era when molecular insights have theranostic value that can outperform the limitations of standard therapeutic options, molecular tumor boards to determine the appropriateness of novel therapies could help patients access breakthrough approaches whose availability is hampered by the regulatory mechanism, but which are nevertheless readily available and tailor made for their individual cancers.

## Consent

Written informed consent was obtained from the patient for publication of this case report and the accompanying images. A copy of the written consent is available for review by the Editor-in-Chief of this journal.
